# Lifestyle and Pharmacological Interventions to Prevent Anthracycline-Related Cardiotoxicity in Cancer Patients

**DOI:** 10.3390/jcdd12060212

**Published:** 2025-06-04

**Authors:** Luigi Spadafora, Francesca Maria Di Muro, Chiara Intonti, Ludovica Massa, Mauro Monelli, Roberto Franco Enrico Pedretti, Edvige Palazzo Adriano, Pasquale Guarini, Gaia Cantiello, Marco Bernardi, Federico Russo, Stefano Cacciatore, Pierre Sabouret, Michele Golino, Giuseppe Biondi Zoccai, Francesca Romana Zimatore, Laura Adelaide Dalla Vecchia

**Affiliations:** 1 Department of Medical-Surgical Sciences and Biotechnologies, Sapienza University of Rome, 04100 Latina, Italy; gaiacantiello@live.it (G.C.); marco.bernardi23@gmail.com (M.B.); gbiondizoccai@gmail.com (G.B.Z.); 2 Division of Cardiology and Interventional Cardiology, Santa Maria Goretti Hospital, 04100 Latina, Italy; 3 Department of Medicine, Surgery and Dentistry, University of Salerno, Baronissi, Salvador Allende Street 43, 84081 Salerno, Italy; fdimuro94@gmail.com; 4 Department of Clinical, Internal Medicine, Anesthesiology and Cardiovascular Sciences, Sapienza University of Rome, 00161 Rome, Italy; chiaraintonti94@gmail.com; 5 Department of Biomedical Sciences and Community Health, University of Milan, Via Mangiagalli 31, 20133 Milan, Italy; ludovica.massa@unimi.it; 6 Department of Cardiology, IRCCS Istituti Clinici Scientifici Maugeri, 20138 Milan, Italy; mauro.monelli@icsmaugeri.it (M.M.); edvige.palazzoadriano@icsmaugeri.it (E.P.A.); 7 School of Medicine and Surgery, University of Milano Bicocca, 20216 Milan, Italy; roberto.pedretti@unimib.it; 8 Cardiology Unit, Hospital of Erba, 22036 Erba, Italy; 9 UO Cardiologia, Clinica Sanatrix, 80127 Naples, Italy; guarini@iol.it; 10 Centro Studi SICOA, 80127 Naples, Italy; 11 Department of Clinical and Molecular Medicine, Sapienza University of Rome, 00185 Rome, Italy; fedrusso1@gmail.com; 12 Department of Geriatrics, Orthopedics, and Rheumatology, Università Cattolica del Sacro Cuore, L.go F. Vito 1, 00168 Rome, Italy; stefanocacciatore@live.it; 13 Fondazione Policlinico Universitario “Agostino Gemelli” IRCCS, L.go A. Gemelli 8, 00168 Rome, Italy; 14 Heart Institute and Action Group, Pitié-Salpétrière, Sorbonne University, 75013 Paris, France; cardiology.sabouret@gmail.com; 15 VCU Pauley Heart Center, Virginia Commonwealth University, Richmond, VA 23298, USA; micheleg1390@gmail.com; 16 Maria Cecilia Hospital, GVM Care & Research, 48033 Cotignola, Italy; 17 Cardiovascular Diseases Residency Program, Azienda Ospedaliera Universitaria Integrata di Verona, 37126 Verona, Italy; francescazimatore@yahoo.it

**Keywords:** anthracyclines, anthracycline-induced cardiotoxicity, prevention, exercise, pharmacological strategies, cardio-oncology, cardiac rehabilitation, cardiac pre-habilitation

## Abstract

Anthracyclines remain a cornerstone of cancer therapy but are associated with a significant risk of cardiotoxicity, which can lead to overt heart failure. The risk is modulated by cumulative dose, pre-existing cardiovascular disease, and patient-specific factors. As cancer survival improves, the long-term cardiovascular consequences of anthracycline exposure have become a growing concern, underscoring the need for effective preventive strategies. This narrative review examines lifestyle and pharmacological interventions aimed at mitigating anthracycline-induced cardiotoxicity. Evidence suggests that structured exercise programs and antioxidant-rich diets may enhance cardiovascular resilience, while beta-blockers, renin-angiotensin system inhibitors, and dexrazoxane remain central pharmacological options. Emerging therapies, including sodium-glucose co-transporter 2 inhibitors and sacubitril/valsartan, show promise but require further investigation. A comprehensive approach that integrates lifestyle modifications with pharmacological strategies within a multidisciplinary cardio-oncology framework may provide optimal protection, improving long-term cardiovascular outcomes in cancer patients receiving anthracyclines.

## 1. Introduction

Anthracyclines, including doxorubicin, daunorubicin, and epirubicin, are among the most effective chemotherapeutic agents used for several malignancies, including breast cancer, lymphomas (Hodgkin and aggressive subtypes of non-Hodgkin), and acute leukemias [1]. However, their use is limited by their potential to cause anthracycline-induced cardiotoxicity (AIC), which can manifest as left ventricular (LV) dysfunction, heart failure (HF), or other cardiovascular complications [1,2]. The risk of AIC increases with cumulative dose exposure but is also influenced by patient-specific factors, including pre-existing cardiovascular disease, age, sex, and genetic predisposition [3]. In long-term cancer survivors, AIC is a leading cause of non-cancer-related mortality, highlighting the need for early prevention and intervention strategies [4].

Recent advancements in cardio-oncology have led to a growing emphasis on cardioprotective measures aimed at minimizing the impact of anthracyclines on cardiac function [2,5]. These include lifestyle modifications, such as exercise and dietary strategies, and pharmacological approaches, including beta-blockers, renin-angiotensin system inhibitors, and emerging therapies like sodium-glucose co-transporter 2 (SGLT2) inhibitors [3,4]. Additionally, multidisciplinary cardio-oncology teams have successfully integrated these strategies into routine cancer care, enabling risk stratification and personalized follow-up plans [2].

This review provides a comprehensive analysis of lifestyle and pharmacological interventions to prevent AIC, exploring their mechanisms, clinical evidence, and practical application. By highlighting the synergy between exercise, nutrition, and medical therapy, we aim to inform clinicians on the most effective strategies to preserve cardiac health in cancer patients undergoing anthracycline-based treatments.

## 2. Epidemiology of Anthracycline-Induced Cardiotoxicity

Anthracyclines are widely used in oncology for the treatment of both solid and hematologic malignancies, particularly breast cancer, lymphomas (both Hodgkin and non-Hodgkin), and leukemias [6]. AIC can present in different forms, ranging from subclinical myocardial dysfunction to overt HF, with incidence rates varying depending on the patient population, cumulative dose, and presence of predisposing risk factors [2,3].

### 2.1. Incidence and Prevalence in Adult and Pediatric Populations

The incidence of AIC in adults varies between 3% and 20%, depending on factors such as total anthracycline exposure, patient comorbidities, and concurrent administration of other cardiotoxic agents [1]. Cardiotoxicity can manifest acutely during or shortly after treatment, though this is relatively rare, occurring in less than 1% of cases [7,8]. More commonly, patients develop early-onset chronic cardiotoxicity within the first year after treatment or late-onset chronic cardiotoxicity, which may remain subclinical for years before progressing to symptomatic heart failure [9,10].

In pediatric cancer patients, the risk of AIC is even more pronounced [11]. Studies report a broad incidence range, from 2% to 65%, largely influenced by cumulative dose, radiation exposure, and individual susceptibility [12,13]. Given that the pediatric myocardium is still developing, children treated with anthracyclines face a heightened lifetime risk of progressive cardiac dysfunction. Survivors of childhood cancers often experience a gradual decline in LV function, emphasizing the importance of long-term cardiovascular monitoring in this population [4].

### 2.2. Anthracycline Use Across Different Malignancies

In breast cancer, anthracyclines have historically been a mainstay of treatment, particularly for high-risk and triple-negative disease [3,12]. However, evolving strategies now consider dose reduction or omission in specific subgroups. For instance, in human epidermal growth factor receptor 2 (HER2)-positive early-stage breast cancer, emerging evidence suggests that anthracycline-free regimens may offer similar efficacy while minimizing cardiotoxic risk [4]. Despite these efforts, anthracyclines remain a key component in the treatment of aggressive forms of breast cancer.

In hematologic malignancies, including acute leukemias and lymphomas (Hodgkin and aggressive subtypes of non-Hodgkin), anthracyclines play a central role in the induction and consolidation chemotherapy [3,14]. Their potent cytotoxic activity is critical for disease control, although the cumulative cardiotoxic burden in these patients remains a concern, particularly for those undergoing multiple cycles of treatment [3].

Gynecologic cancers, particularly advanced or recurrent ovarian and endometrial cancers, also rely on anthracycline-based chemotherapy in selected cases [15]. While newer targeted therapies have improved outcomes, anthracyclines continue to be used in refractory disease settings, contributing to the overall burden of cardiotoxicity in women with gynecologic malignancies.

### 2.3. Cumulative Dose and Cardiotoxicity Thresholds

The relationship between anthracycline dose and cardiotoxicity is well established [3,13]. For doxorubicin, the most widely used anthracycline, the incidence of cardiotoxicity rises significantly with cumulative exposure [2]. At a dose of 400 mg/m^2^, the estimated risk is around 5%, increasing to 26% at 550 mg/m^2^ and nearly 50% at 700 mg/m^2^ [3]. Due to these risks, current guidelines recommend limiting cumulative doxorubicin exposure to 400–450 mg/m^2^ in most patients [2,8]. Other anthracyclines, such as epirubicin and daunorubicin, exhibit similar dose-related toxicity but with slightly different thresholds [3,16].

### 2.4. Identification of High-Risk Populations

Several patient subgroups are particularly vulnerable to AIC. Older adults are at increased risk due to reduced cardiac reserve and heightened susceptibility to oxidative stress and inflammation. In pediatric cancer survivors, incomplete myocardial development at the time of treatment predisposes them to long-term cardiac complications, with many experiencing a progressive decline in systolic function that can manifest decades later.

Patients with pre-existing cardiovascular disease, including hypertension, diabetes, or coronary artery disease, have an amplified risk of AIC due to the additive stress on the myocardium. Similarly, individuals receiving concurrent cardiotoxic therapies, such as trastuzumab or thoracic radiation, face a compounded risk of developing cardiac dysfunction.

Sex-related differences have also been observed, with female patients appearing more susceptible to anthracycline-induced cardiotoxicity than males. The underlying mechanisms are not fully understood but may involve hormonal influences, genetic factors, and differences in myocardial structure. Genetic predisposition, including polymorphisms affecting oxidative stress pathways and drug metabolism, has also been implicated in interindividual variability in AIC risk.

### 2.5. Long-Term Outcomes and Healthcare Burden

Anthracycline-induced HF can develop during treatment or years after therapy completion [1]. Subclinical LV dysfunction is common in long-term cancer survivors, and without appropriate monitoring, many patients progress to symptomatic HF [10,17]. The presence of AIC significantly worsens survival outcomes, with five-year mortality rates substantially higher in patients who develop anthracycline-related HF compared to those without cardiac complications [8].

The long-term management of AIC places a considerable burden on healthcare systems [4]. Routine cardiac surveillance, including serial echocardiography, biomarker assessments such as troponins and N-terminal pro-b-type natriuretic peptide (NT-proBNP), and, in select cases, cardiac magnetic resonance imaging (MRI), is necessary to detect early myocardial changes [18]. In patients who develop HF, treatment requires lifelong pharmacologic management, frequent hospitalizations and, in advanced cases, consideration of device therapy or heart transplantation [4].

Efforts to mitigate the impact of AIC include dose modifications, the use of liposomal anthracyclines to reduce myocardial exposure, and the cardioprotective agent dexrazoxane, which chelates iron and reduces anthracycline-induced oxidative stress, with efficacy in selected high-risk patients [6,19].

## 3. Mechanisms of Anthracycline-Induced Cardiotoxicity

The mechanisms underlying AIC are complex and multifactorial, involving oxidative stress, mitochondrial dysfunction, alterations in calcium homeostasis, and dysregulation of cell death pathways [3,20]. While anthracyclines exert their anticancer effects primarily through topoisomerase II (Top2) inhibition, their off-target effects in cardiomyocytes lead to cumulative and often irreversible myocardial damage [3,7].

### 3.1. Oxidative Stress and Mitochondrial Dysfunction

One of the most recognized mechanisms of AIC is excessive oxidative stress, driven by an imbalance between reactive oxygen species (ROS) production and antioxidant defenses [21,22]. Anthracyclines have a high affinity for cardiolipin, a phospholipid abundant in the inner mitochondrial membrane, facilitating their accumulation in mitochondria [22,23]. Within these organelles, anthracyclines undergo redox cycling via interactions with nicotinamide adenine dinucleotide phosphate (NADPH) oxidase (Nox), nitric oxide synthase (NOS), and electron transport chain components, leading to the generation of superoxide (O^2−^) and hydrogen peroxide (H_2_O_2_) [23,24]. This disrupts mitochondrial function by impairing complexes I and IV of the respiratory chain in the mitochondria, resulting in further ROS production, energy depletion, and metabolic reprogramming [25].

Iron metabolism also plays a role in amplifying oxidative stress [26]. Anthracyclines interfere with iron homeostasis by altering iron regulatory protein 1 (IRP-1), increasing transferrin receptor expression and reducing ferritin synthesis [26]. This results in elevated intracellular free iron, which promotes hydroxyl radical formation via the Fenton reaction [26,27]. Furthermore, mitochondrial iron accumulation due to the downregulation of the ATP-binding cassette (ABC)-B8 transporter exacerbates ROS generation and mitochondrial injury [26,28].

Cardiomyocytes are particularly vulnerable to oxidative damage due to their lower antioxidant capacity compared to other tissues [29]. Anthracycline exposure further depletes key antioxidant enzymes such as superoxide dismutase (SOD) and glutathione peroxidase (GPX), and impairs vitamin C uptake through downregulation of sodium-dependent vitamin C transporters [22,29]. The resulting oxidative stress contributes to lipid peroxidation, protein oxidation, and mitochondrial DNA damage, all of which compromise cardiac function [21].

### 3.2. Disruption of Calcium Homeostasis

Anthracyclines also interfere with calcium handling, a key regulator of myocardial contractility [27]. They upregulate L-type calcium channels by increasing calcium voltage-gated channel subunit alpha1C (CACNA1C) expression, leading to excessive calcium influx [27,30]. Simultaneously, they activate ryanodine receptor 2 (RyR2) channels, promoting uncontrolled calcium release from the sarcoplasmic reticulum (SR) into the cytosol [27,31]. At the same time, sarco/endoplasmic reticulum Ca^2+^-ATPase (SERCA2A) function is impaired by oxidative modifications, reducing the efficiency of calcium reuptake into the SR [27]. This results in sustained cytosolic calcium overload, which disrupts excitation-contraction coupling and contributes to cardiomyocyte dysfunction [27].

Excess cytosolic calcium is also sequestered by mitochondria, where it forms calcium phosphate complexes, leading to structural damage and impaired ATP synthesis. Prolonged mitochondrial calcium accumulation can trigger the opening of the mitochondrial permeability transition pore (mPTP), causing a loss of membrane potential, energy collapse, and eventual cell death [27].

### 3.3. Activation of Cell Death Pathways

AIC is characterized by the activation of multiple cell death pathways, including apoptosis, autophagy, and pyroptosis [32,33,34].

Apoptosis, the most extensively studied form of cell death in AIC, is activated through both intrinsic and extrinsic pathways [35]. The intrinsic pathway involves mitochondrial dysfunction, upregulation of pro-apoptotic proteins such as BAX and BAK, and the release of cytochrome c, which triggers caspase-mediated apoptosis [22]. This process is exacerbated by p53 overexpression, c-Jun N-terminal kinase/mitogen-activated protein kinase (JNK/MAPK) activation, and phosphoinositide 3-kinase/protein kinase B (PI3K/Akt) inhibition [35].

In addition to apoptosis, anthracyclines influence autophagy, a cellular process responsible for degrading damaged organelles and maintaining energy homeostasis [33]. The role of autophagy in AIC remains controversial, with evidence suggesting both protective and detrimental effects [34,36]. While autophagy activation through AMP-activated protein kinase (AMPK) and p53 pathways may serve as a compensatory mechanism to mitigate oxidative damage, excessive autophagosome accumulation due to lysosomal dysfunction can lead to cell death [37]. Anthracyclines inhibit lysosomal acidification and impair the fusion of lysosomes with autophagosomes, leading to the accumulation of undegraded cellular debris and further promoting oxidative stress [38].

Pyroptosis, a highly inflammatory form of programmed cell death, has also been implicated in AIC [29]. This process is mediated by the NOD-like receptor pyrin domain-containing 3 (NLRP3) inflammasome, which is activated in response to mitochondrial dysfunction, ROS accumulation, and intracellular danger signals [39,40]. Once activated, NLRP3 triggers caspase-1-mediated cleavage of gasdermin D, forming membrane pores that lead to cell lysis and the release of pro-inflammatory cytokines such as interleukin(IL)-1β and IL-18 [39]. Anthracyclines can also induce pyroptosis through Bcl-2/adenovirus E1B 19-kDa-interacting protein 3 (BNIP3)-mediated caspase-3 activation, which promotes gasdermin E cleavage and subsequent membrane disruption [41]. This sustained inflammatory response contributes to myocardial remodeling and accelerates the progression to HF.

### 3.4. DNA Damage and Epigenetic Modifications

Beyond oxidative stress and cell death pathways, anthracyclines induce direct DNA damage, which plays a critical role in their cardiotoxic effects [42]. While their antitumor efficacy relies on topoisomerase IIα (Top2α) inhibition in cancer cells, they primarily target topoisomerase IIβ (Top2β) in cardiomyocytes [42,43]. The formation of a ternary complex between anthracyclines, Top2β, and DNA leads to double-strand breaks, triggering p53 activation and upregulation of pro-apoptotic genes such as NOXA (Phorbol-12-myristate-13-acetate-induced protein 1) and PUMA (p53 upregulated modulator of apoptosis) [44]. This promotes Bcl-2-associated X protein/Bcl-2 antagonist/killer (BAX/BAK)-mediated mitochondrial apoptosis while simultaneously inhibiting mitophagy, leading to the accumulation of dysfunctional mitochondria and exacerbating oxidative stress [45].

Emerging evidence suggests that anthracyclines also induce epigenetic modifications that may contribute to long-term cardiotoxicity. These include DNA hypomethylation via downregulation of DNA methyltransferase 1 (DNMT1) and histone modifications through upregulation of histone deacetylase 6 (HDAC6) [46]. Additionally, anthracyclines alter microRNA expression, though the functional consequences of these changes require further investigation.

### 3.5. Synergistic Effects with Other Cancer Therapies

The cardiotoxic effects of anthracyclines are often exacerbated when used in combination with other cancer therapies. The HER2 inhibitor trastuzumab, for example, significantly increases the risk of cardiac dysfunction when administered sequentially or concurrently with anthracyclines [47]. Similarly, thoracic radiation therapy induces endothelial damage, fibrosis, and microvascular dysfunction, which can further amplify anthracycline-related cardiotoxicity [4]. The pro-inflammatory state associated with cancer itself may also contribute to enhanced cardiac vulnerability, adding another layer of complexity to the pathophysiology of AIC [48].

## 4. Lifestyle Interventions for Prevention

The development of cancer therapy-related cardiac dysfunction (CTRCD) is shaped by an interplay of patient-specific and drug-specific factors [3]. Of particular importance is the dose-dependent relationship between cumulative drug exposure and the risk of HF. This trajectory can be modulated by several variables: protective factors, such as cardio-protective strategies or liposomal drug formulations, may shift the risk curve rightward, reducing susceptibility, while factors like genetic predisposition, advanced age, or pre-existing cardiovascular risk factors may shift it leftward, and increasing vulnerability even at lower doses [49,50]. These dynamics underscore the need for comprehensive and personalized risk assessment in patients undergoing anthracycline-based therapies [16,51].

Recent guidelines recommend employing the Heart Failure Association-International Cardio-Oncology Society (HFA-ICOS) Risk Assessment Tool, which considers cardiovascular risk factors, clinical history, cancer type, and treatment details [2]. This tool, calculated by the treating oncologist or cardiologist, enables precise stratification of patients into low- or high-risk categories for developing clinical or subclinical HF and facilitates tailored primary or secondary prevention strategies, which may include lifestyle interventions with or without pharmacological measures [8,12]. Crucially, while the latter have not demonstrated significant benefit in low-risk patients undergoing anthracycline-based chemotherapy, they remain essential for those classified as moderate or high risk.

**Figure 1:**
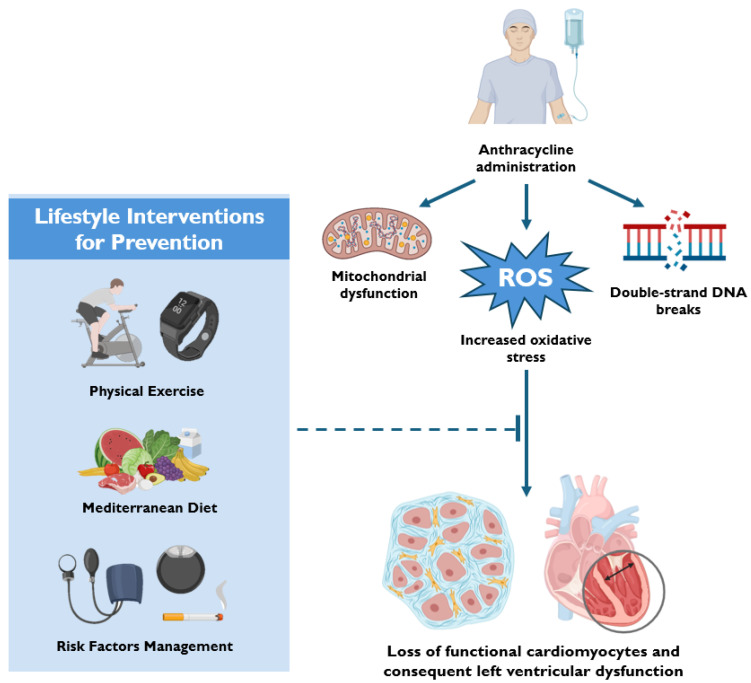
Primary components of lifestyle interventions and their key mechanisms of action. Abbreviations. DNA: deoxyribonucleic acid; ROS: reactive oxygen species.

Lifestyle interventions consist of three main pillars: physical exercise (PE), nutrition, and cardiovascular risk factor management (Figure 1). Among these, PE has emerged as a cornerstone strategy and is now a key recommendation in the latest clinical guidelines [2]. The pathophysiological rationale for PE is multifaceted and comes from its proven efficacy in cardiovascular prevention, pre-habilitation, and rehabilitation in non-cancer populations [52,53].

At a cellular level, preclinical evidence demonstrates that PE reduces anthracycline accumulation, preserves myosin heavy-chain integrity, and mitigates oxidative stress [54,55,56]. These cardioprotective effects persist regardless of PE type, intensity, or timing. Additionally, PE modulates critical cellular pathways, including apoptosis, autophagy, and lysosomal signaling, and enhances myocardial tissue turnover by stimulating cardiomyocyte progenitors and modulating calcium signaling [57,58,59].

At a clinical level, these mechanisms extend beyond the cardiac muscle to influence the entire cardiovascular system as an integrated entity, comprising the lungs, diaphragm, cerebrovascular and peripheral vasculature, nervous system, and metabolic pathways. Indeed, PE promotes cardiomyocyte adaptation and growth, partially counteracting the sarcopenic effects of cancer treatments and contributing to an improved cardiometabolic profile [60]. Enhanced cardiovascular reserve through increased peak oxygen consumption (VO_2_peak) is another key benefit, driven by improved endothelial function, autonomic regulation, and cardiac perfusion [15,61]. Finally, targeted PE also strengthens the diaphragm, lowers vascular resistance, and normalizes calcium-handling proteins, as observed in cardiac recovery programs for HF, collectively enhancing cardiovascular performance [62,63].

Once the impact of PE on cardiovascular function and recovery after anthracycline administration is established, the next critical question is which type of PE offers the greatest benefit and when it should be implemented. Aerobic and resistance PE have shown complementary benefits: aerobic exercise improves cardiorespiratory fitness, while resistance training preserves lean muscle mass, a key factor linked to better survival and outcomes in cancer patients [64,65]. Reflecting this, guidelines from the American College of Sports Medicine (ACSM) and the American Cancer Society (ACS) recommend combining more than 2 h of moderate-intensity or 75 min of high-intensity aerobic PE per week with at least two resistance training sessions [66]. This recommendation has also been adopted into the multidisciplinary Cardio-Oncology Rehabilitation (CORE) framework introduced by the American Heart Association (AHA) in 2019 [67]. Knowledge about newer, nontraditional PE modalities, such as high-intensity interval training (HIIT) and circuit-based exercises, remains limited. Early studies suggest that HIIT may improve endothelial function and slow the progression of atherosclerosis by increasing levels of metalloproteinase [68]. However, concerns about its feasibility and safety in cancer patients persist, highlighting the need for further investigation.

When considering the optimal timing for PE implementation, evidence indicates that PE provides positive effects at any point along the cancer care continuum compared to sedentary behavior [69]. Importantly, as demonstrated by Kang et al., initiating exercise either before or during anthracycline administration may enhance or maintain survivors’ baseline functional capacity, creating a buffer against the inevitable treatment-related decline and helping to prevent crossing into the “disability threshold.” Conversely, starting exercise after treatment may assist in lifting survivors out of the “disability threshold” [70]. PE as pre-habilitation, particularly when initiated immediately after diagnosis, may offer significant advantages: patients are typically in better overall health status at this early stage, making them more likely to adhere to higher-volume and higher-intensity training regimens [71]. On the other hand, the prescription of PE during anthracycline treatment raises important considerations regarding safety and feasibility. Treatment-related side effects, such as nausea, dizziness, or fatigue can hinder adherence to training programs. However, a study by Shephard, R.J. et al. demonstrated that improvements in cardiorespiratory fitness, reflected by significantly higher VO_2_ peak values in patients engaging in PE, were maintained despite an average 20% reduction in exercise volume caused by treatment-related side effects [72]. Feasibility is further challenged by the demands of cancer care, including intensive treatment schedules and frequent medical appointments, which often leave limited time for structured exercise interventions. Addressing these logistical barriers is essential to fully realize the potential benefits of PE during treatment. Finally, PE following cancer treatment capitalizes on the critical transition from cancer survival to overall health improvement, serving as a phase for patient management and education. To this end, programs such as LIVESTRONG^®^ at the YMCA have been introduced as innovative frameworks to support cancer survivors and their families. This initiative provides a structured 12-week physical activity program in a safe and supportive environment, designed to enhance physical, emotional, and mental well-being. However, while this program represents a pioneering effort, data on its specific impact on patients undergoing anthracycline therapy remain limited, underscoring the need for further comprehensive investigation.

Beyond PE, nutrition and cardiovascular risk factor management are essential in reducing CTRCD risk. A scoping review of seven studies by Stephenson et al. highlighted the beneficial effects of various oral supplements, including Coenzyme Q10 (CoQ10), vitamin E, levocarnitine, ginseng, alpha-lipoic acid, vitamin D, and multiflora honey, as well as adherence to a Mediterranean diet [73]. These interventions demonstrated positive effects, such as preserved LV ejection fraction (EF), reductions in cardiac biomarkers (e.g., troponin and creatine kinase-MB), decreased oxidative stress and inflammatory markers (e.g., tumor necrosis factor-α, IL-6), improved metabolic parameters, enhanced body composition, and better quality of life. CoQ10, in particular, is essential for energy production in cardiac tissues due to its role in aerobic respiration and cellular metabolism [74]. Reduced CoQ10 levels have, in fact, been linked to cardiomyopathies and chronic cardiac dysfunction, making its supplementation of particular therapeutic interest, especially as an adjunct in HF medical treatments [75,76,77]. The observed improvements in LVEF are likely attributable to the ability of CoQ10 to inhibit plasma low-density lipoprotein oxidation, enhance endothelial function, and support myocardial bioenergetics [78]. Vitamin E is a potent lipid-soluble antioxidant that helps preserve cardiovascular health by protecting polyunsaturated cellular membrane lipids against free radical-induced damage and modulating inflammatory markers, including C-reactive protein (CRP) and IL-6. Vitamin D exhibits complementary properties, with its active metabolite, 1,25-dihydroxyvitamin D, reducing IL-6 and other inflammatory mediators via p38 signaling pathways while also decreasing ROS and mitigating mitochondrial damage without compromising the therapeutic efficacy of anthracycline-based treatments [79,80,81]. These nutritional elements, along with polyphenols and omega-3 fatty acids, are part of the Mediterranean diet, which includes fruits, vegetables, whole grains, legumes, nuts, olive oil, moderate amounts of fish and poultry, and limited alcohol consumption [82]. This dietary pattern has been shown to enhance therapeutic responsiveness and reduce the risk of chemotherapy-induced cardiac damage, as demonstrated in a preclinical study involving mice with triple-negative breast cancer. The same study also revealed that while mice on a Western diet experienced significant bone loss due to the anticancer treatment, increasing their risk of fractures, those on a Mediterranean diet appeared to be protected [83]. The management of modifiable risk factors constitutes the third essential pillar of lifestyle prevention within the multidisciplinary CORE approach and is strongly endorsed by the latest cardio-oncology guidelines [2]. This includes optimizing blood pressure control, promoting smoking cessation, and implementing targeted interventions for diabetes and lipid management, in alignment with the 2021 guidelines from the European Society of Cardiology on cardiovascular disease prevention [84].

Recently, the International Cardio-Oncology Society (ICOS) established the Cardio-Oncology Rehabilitation and Exercise (CORE) working group to enhance the integration of structured exercise and cardiovascular risk management into routine cancer care [85]. The ICOS-CORE initiative emphasizes the importance of multidisciplinary interventions, leveraging principles from cardiac rehabilitation to improve the cardiovascular health of cancer survivors [85]. This model is designed to address key gaps in cardio-oncology by standardizing exercise prescriptions, optimizing risk factor management, and promoting adherence to lifestyle modifications [85]. Despite its potential benefits, the widespread implementation of CORE remains limited by challenges such as insufficient reimbursement policies and variability in program availability. Nevertheless, emerging data support its effectiveness in reducing cardiovascular toxicity and improving long-term outcomes in cancer patients undergoing anthracycline-based treatments.

## 5. Pharmacological Strategies for Prevention

Pharmacological strategies for the primary prevention of CTRCD include but are not limited to well-established neurohormonal antagonists—such as angiotensin-converting enzyme (ACE)-inhibitors (ACE-I), angiotensin II receptor blockers (ARBs), and beta-blockers—along with statins, dexrazoxane and promising emerging therapies currently under investigation. Principal randomized controlled trials evaluating established therapies for anthracycline-induced cardiotoxicity are summarized in Table 1.

The use of ACE-Is or ARBs is supported by the central role of the renin-angiotensin-aldosterone system in the pathogenesis of anthracycline-induced cardiotoxicity [97]. Anthracyclines, particularly doxorubicin, elevate angiotensin-II levels and ACE activity up to threefold, directly contributing to myocardial damage [43,97]. Targeting these enzymes has demonstrated cardioprotective benefits, as evidenced by preclinical studies, with zofenopril demonstrating superior efficacy attributed to its sulfhydryl group, which confers ROS scavenger activity and higher affinity for cardiomyocytes [98]. Similarly, small randomized controlled trials (RCTs) have reported positive outcomes across various cancer types [87,99].

Beta-blockers also hold promise in mitigating cardiotoxicity through neurohormonal modulation, heart rate control, and arrhythmia prevention, with carvedilol and nebivolol offering additional benefits due to their antioxidant properties. However, clinical evidence remains mixed. A small RCT by Kaya et al. suggested prophylactic nebivolol could prevent LV dilatation and functional impairment associated with chemotherapy regimens containing adriamycin or epirubicin for breast cancer [88]. Conversely, the larger (Carvedilol Effect in Preventing Chemotherapy-Induced Cardiotoxicity) CECCY trial reported that carvedilol (50 mg) did not significantly prevent LVEF reduction at six months, although it effectively reduced troponin elevation and the incidence of diastolic dysfunction [89].

When ACEIs/ARBs and beta blockers are combined for their synergistic effect on the neurohormonal axis, the clinical evidence becomes increasingly mixed. For instance, the PRADA trial (Prevention of Cardiac Dysfunction During Adjuvant Breast Cancer Therapy), which employed a 2 × 2 factorial design to assess candesartan cilexetil, metoprolol succinate, their combination, or placebo in early breast cancer patients receiving adjuvant epirubicin therapy, found no significant benefits of the combined treatment on LVEF, global longitudinal strain (GLS), or left ventricular end-systolic diameter (LVESD). However, candesartan alone attenuated declines in GLS and LV end-diastolic volume, suggesting a potential role in monotherapy [91,92]. On the other hand, the OVERCOME trial (preventiOn of left Ventricular dysfunction with Enalapril and caRvedilol in patients submitted to intensive ChemOtherapy for the treatment of Malignant hEmopathies) indicated that concomitant enalapril and carvedilol therapy may prevent LV systolic dysfunction in patients with malignant hemopathies undergoing high-dose chemotherapy, despite the limitation of a small sample size of only 90 patients.

MRAs have also been proposed as an additional therapeutic option. While their role as a cornerstone in HF management is well-established, evidence from murine models suggests that mineralocorticoid receptor suppression may counteract doxorubicin-induced repression of RNA sequencing in isolated cardiac myocytes [100]. However, current clinical evidence is limited to small RCTs, with the ELEVATE trial (Effect of Eplerenone on Left Ventricular Diastolic Function in Women Receiving Anthracyclines for Breast Cancer) failing to demonstrate any critical discrepancy in systolic or diastolic function between six months of eplerenone administration and placebo in patients receiving anthracycline for early or locally advanced breast cancer undergoing [93].

Statins have garnered attention for their pleiotropic effects beyond cholesterol-lowering, including indirect inhibition of small Ras homologous GTPases like Rac1, a critical regulator of NADPH oxidase and Top2, both implicated in anthracycline-induced cardiotoxicity [101,102,103,104]. These properties have sparked significant interest in their potential as cardioprotective agents, leading to their introduction as a Class IIa recommendation for primary prevention in patients at high or very high risk of CTRCD, despite divergent results from the most recent RCTs. The PREVENT (Preventing Anthracycline Cardiovascular Toxicity with Statins) trial failed to demonstrate the effectiveness of daily atorvastatin 40 mg, started before anthracycline therapy and maintained for 24 months, in preventing anthracycline-induced LV dysfunction among 279 patients with breast cancer or lymphoma [94]. Similarly, the smaller SPARE-HF (Statins for the Primary Prevention of Heart Failure in Patients Receiving Anthracycline Pilot Study) trial found no significant benefit of atorvastatin in reducing LVEF decline compared to placebo (0.79%; *p* = 0.34) [95]. In contrast, the STOP-CA (Statins to Prevent the Cardiotoxicity of Anthracyclines) trial observed that, among 300 adults with lymphoma, the same therapeutic approach (atorvastatin 40 mg daily) reduced the risk of cardiac dysfunction over 12 months compared with placebo [96].

A potential explanation for these conflicting results could be the dose-dependent cardiotoxicity of anthracyclines, which may account for the protective effect observed only in the STOP-CA trial, where high-dose anthracyclines were used. Other factors might include the smaller sample sizes in the two neutral trials, both of which also experienced higher dropout rates compared to the STOP-CA trial, as well as differences in overall follow-up duration or the concomitant use of neurohormonal modulators, which may have influenced the outcomes. Given these uncertainties, statins cannot yet be universally recommended as a preventive therapeutic strategy for all patients receiving anthracyclines. Conclusive evidence from larger RCTs is needed to identify the cancer patient subgroups most likely to benefit from statin therapy, as well as to determine the optimal timing, dose and duration of treatment.

## 6. Novel Therapeutic Pharmacological Interventions

Recent research has explored the potential cardioprotective effects of novel pharmacological agents in mitigating anthracycline-induced cardiotoxicity [105]. Preclinical and early-phase clinical studies suggest that SGLT2 inhibitors, glucagon-like peptide-1 receptor agonists (GLP-1 RAs), vericiguat, and sacubitril/valsartan may offer cardioprotective benefits through distinct molecular pathways [106,107].

### 6.1. SGLT2 Inhibitors

SGLT2 inhibitors, initially developed for glycemic control in diabetes, have demonstrated cardioprotective properties in the context of HF and, more recently, in cardio-oncology [107]. Preclinical studies have shown that empagliflozin reduces doxorubicin-induced cardiac dysfunction by preventing mitochondrial dysfunction, inflammation, and oxidative stress in murine models [108]. Furthermore, these agents appear to inhibit the NLRP3 inflammasome pathway, thereby reducing myocardial fibrosis and cardiomyocyte apoptosis [109]. Clinical studies have also indicated a potential role for SGLT2 inhibitors in reducing cardiovascular complications in cancer patients receiving anthracyclines [106,110]. These findings provide a rationale for further clinical investigations evaluating SGLT2 inhibitors as cardioprotective agents in oncologic populations, as suggested by a recent metanalysis [105].

### 6.2. GLP-1 Receptor Agonists

GLP-1 receptor agonists have been investigated for their role in attenuating anthracycline-induced myocardial damage. Preclinical studies indicate that semaglutide ameliorates doxorubicin-induced cardiotoxicity by reducing mitochondrial dysfunction via inhibition of BNIP3 signaling, a key mediator of mitophagy [111]. Similarly, tirzepatide, a dual GLP-1/GIP receptor agonist, has demonstrated protective effects against doxorubicin cardiotoxicity by modulating the PI3K/Akt signaling pathway, which mitigates oxidative stress and inflammation [112].

### 6.3. Vericiguat

Vericiguat, a soluble guanylate cyclase (sGC) stimulator, has shown promise in preclinical models of anthracycline-induced cardiomyopathy. Studies suggest that vericiguat reduces myocardial inflammation and mitochondrial dysfunction via upregulation of the PRKG1/PINK1 pathway, which counteracts oxidative stress and apoptosis [113]. Additionally, its effects on the NLRP3 inflammasome may contribute to both cardioprotection and the attenuation of sarcopenia, which is a common complication in cancer patients undergoing chemotherapy [114].

### 6.4. Sacubitril/Valsartan

Sacubitril/valsartan, a neprilysin inhibitor and angiotensin receptor blocker, has been extensively studied in HF but is now being investigated for its potential in preventing chemotherapy-induced cardiotoxicity [2]. Preclinical models suggest that sacubitril/valsartan attenuates doxorubicin-induced myocardial inflammation, fibrosis, and apoptosis via modulation of the AMPKα-mTORC1 pathway [115]. In addition, clinical trials such as the MAINSTREAM study aim to assess its efficacy in preventing LV dysfunction in breast cancer patients receiving anthracyclines [115,116].

## 7. Role of Multidisciplinary Cardio-Oncology Teams: Integrating Prevention and Management

The integration of cardio-oncology teams into cancer care has become essential for improving the prevention and management of CTRCD, particularly in patients receiving anthracycline-based treatments. These teams typically comprise cardiologists, oncologists, nurses, physiotherapists, nutritionists, and other healthcare professionals working collaboratively to minimize cardiovascular complications while ensuring optimal oncological outcomes. Through a structured approach that incorporates early risk assessment, preventive strategies, and tailored follow-up protocols, multidisciplinary teams help reduce the burden of long-term cardiovascular sequelae in cancer survivors.

## 8. Personalized Strategies Using Risk Stratification Tools

The 2022 European Society of Cardiology (ESC) guidelines on cardio-oncology recommend baseline risk stratification as the foundation for developing strategies to prevent, monitor, and manage CTRCD [2,4,5]. This approach aims to identify patients at risk while minimizing resource utilization for those less likely to develop CTRCD [6].

The HFA-ICOS risk assessment tool, a critical framework in this field, was developed by the Heart Failure Association (HFA) of the European Society of Cardiology (ESC) in collaboration with the International Cardio-Oncology Society (ICOS) [2]. This model aims to provide a structured, evidence-based approach to evaluate cardiovascular risks, enabling better prevention and management of CTRCD. This tool demonstrated a sensitivity of 49.3%, specificity of 87.9%, positive predictive value of 23.3%, negative predictive value of 95.6%, and overall accuracy of 85.3% when predicting the onset of symptomatic or severe/moderate asymptomatic CTRCD in patients classified as low–moderate risk versus high–very high risk [117].

The HFA-ICOS risk assessment tool incorporates a wide range of patient-specific and treatment-specific factors that contribute to cardiotoxicity risk [8]. These include (Table 2):

The HFA-ICOS tool (Table 2), evaluating these multiple risk factors, classifies patients into different categories of risk (low, intermediate, or high) based on their likelihood of developing HF or other cardiac dysfunction during or after cancer treatment [12,14].

The HFA-ICOS risk stratification demonstrated effective differentiation and reliable accuracy in forecasting symptomatic or severe/moderate asymptomatic CTRCD at both 6 and 12 months [118]. This timeframe is crucial for the onset of anthracycline-induced toxicity, emphasizing the value of using this model to guide prevention efforts for high-risk patients.

## 9. Long-Term Follow-Up Protocols

### 9.1. Biomarkers, Imaging, and Lifestyle Reassessment in Survivors

Early identification of CTRCD is crucial for its prevention and treatment due to its unfavorable prognosis [13]. To date, metabolic markers and imaging methods are the primary strategies used for this purpose [10]. Early CTRCD detection can be achieved through a combination of imaging modalities, cardiac biomarkers, and genetic analysis [18]. The baseline assessment of GLS can aid in evaluating the risk for patients presenting with a LVEF between 50% and 59%. A GLS value of less than 16% or a relative change greater than 15% from baseline serve as important risk markers, signaling the potential need for cardioprotective treatment before any decrease in LVEF occurs [17]. Noninvasive metrics such as myocardial work and LV-arterial coupling, which assess chamber stiffness and arterial load, have established prognostic roles in the general HF population. While these metrics show promise for detecting changes in diastolic performance following anthracycline exposure, additional studies in cancer populations are needed [119]. The right ventricle is also vulnerable to anthracycline-induced damage, and three-dimensional echocardiography and strain imaging can be useful in evaluating right ventricular function in these patients. Although cardiac magnetic resonance (CMR) is not routinely used in routine cancer patient monitoring due to availability and cost-related considerations, its high reproducibility in estimating LVEF, biventricular volume, and mass, alongside its unique capacity for tissue characterization, has led to increased utilization in clinical trials. CMR can detect microvascular obstruction, tissue iron overload, and diffuse interstitial fibrosis through native T1 mapping and calculation of the extracellular volume fraction, while myocardial edema can be quantified via T2 mapping [120]. CMR is particularly useful in clinical practice when echocardiography is limited or when clinical suspicion of myocarditis arises. Moreover, CMR plays a role in diagnosing pericardial disease, which is common among patients exposed to anthracycline therapy [121]. High-sensitivity cardiac biomarkers, such as troponins and natriuretic peptides (NPs), have significantly enhanced the early detection of cardiac damage, with troponins demonstrating predictive and prognostic value in assessing cardiotoxicity. While NPs have been extensively studied in predicting CTRCD, interpretation of their effects must take into account confounding factors such as renal function, body weight, and the use of angiotensin receptor neprilysin inhibitors. Evaluating cardiac biomarkers prior to chemotherapy can help stratify baseline cardiotoxicity risk, but these findings must be integrated with imaging and electrocardiographic data. Although the exact thresholds for defining clinically significant events are still being determined, troponins exhibit a high negative predictive value, allowing for the identification of low-risk patients.

Myeloperoxidase (MPO) is an enzyme predominantly found in the azurophilic granules of myeloid cells and is abundant in neutrophils. It is released when neutrophils are activated. Elevated levels of MPO are observed in the body when the myocardium is damaged, especially with anthracycline treatment, as oxidative stress plays a central role in anthracycline-induced cardiotoxicity. MPO acts as both a marker and mediator of inflammation and oxidative stress. Higher MPO levels in peripheral blood, both before and after the first doxorubicin treatment, are associated with an increased risk of cardiotoxicity in breast cancer patients [122].

Genetic variations can significantly affect an individual’s susceptibility to anthracycline-induced cardiotoxicity. Key genes involved include those linked to hereditary cardiomyopathies (e.g., titin mutations), those responsible for ROS production and detoxification, and genes involved in drug metabolism and transport. Regulatory microRNAs also play a crucial role. Genetic alterations may influence the cellular transport and clearance of anthracyclines, increasing cardiotoxicity. In mitochondria, anthracyclines generate superoxide anions, and polymorphisms in NADPH oxidase subunits can worsen ROS production. Emerging evidence suggests that mitochondrial function assessed in peripheral blood-derived cells may reflect systemic mitochondrial alterations, potentially offering a non-invasive window into cardiovascular health [123]. In the context of anthracycline-induced cardiotoxicity, peripheral mitochondrial assessment could, in the future, be explored as a surrogate marker to monitor early subclinical cardiac dysfunction and guide long-term follow-up strategies [123]. Genetic variants linked to inherited cardiomyopathies may further increase vulnerability to anthracycline-induced damage [124].

### 9.2. Integration of Cardio-Protective Strategies into Clinical Workflows

Integrating cardio-protective strategies into the clinical workflow is essential in ensuring that cancer survivors are receiving comprehensive care that includes both oncological and cardiovascular management [3,4]. Strategies to achieve this integration include multidisciplinary teams that provide personalized care for cancer patients at risk of cardiovascular complications, with regular communication between oncology and cardiology teams, and shared decision-making involving both oncology and cardiology allows for tailored interventions that balance cancer treatment goals and heart health [2]. The clinicians treating the patient should use risk prediction models like the HFA-ICOS risk assessment tool that help the team evaluate cardiotoxicity risk at the time of diagnosis and throughout cancer treatment. The risk stratification should be reassessed periodically during the survivor’s care to adjust treatment or preventive measures based on changes in the patient’s condition. Cardiotoxicity prevention may involve the use of cardioprotective agents such as ACE inhibitors, beta-blockers, or liposomal drug formulations to reduce the risk of cardiac damage. The use of statins, dexrazoxane, and other emerging therapies may be considered for patients at high risk for cardiotoxicity.

## 10. Conclusions and Future Directions

Anthracycline-induced cardiotoxicity remains a key challenge in oncology, requiring a proactive and multidisciplinary approach. Advances in risk stratification, biomarker surveillance, and cardioprotective therapies have improved early detection and management, yet optimizing prevention strategies remains a priority. Emerging pharmacological interventions, including SGLT2 inhibitors, GLP-1 receptor agonists, vericiguat, and sacubitril/valsartan, hold promise, but large-scale clinical trials are needed to confirm their efficacy. Likewise, genetic and biomarker-driven risk models may refine patient selection for personalized prevention. Multidisciplinary cardio-oncology teams are essential to integrating cardiovascular care into oncology workflows. Standardized surveillance protocols, including advanced imaging and risk assessment tools like HFA-ICOS, will further improve outcomes. Future efforts should focus on precision medicine and risk stratification, ensuring that cancer patients survive and maintain long-term cardiovascular health. Bridging cardiology and oncology is the new standard of care.

## Figures and Tables

**Table 1:** Principal randomized controlled trials evaluating established therapies for anthracycline-induced cardiotoxicity. Abbreviations. ACE-i: angiotensin-converting enzyme inhibitors; ALL: acute lymphoblastic leukemia; AML: acute myeloid leukemia; ARBs: angiotensin II receptor blockers; CTRCD: cancer therapy-related cardiac dysfunction; EDV: end-diastolic volume; ESV: end-systolic volume; GLS: global longitudinal strain; LVEDD: left ventricular end-diastolic diameter; LVEF: left ventricular ejection fraction; LVESD: left ventricular end-systolic diameter; MM: multiple myeloma; MRAs: mineralocorticoid receptor antagonists.

Study	Study Design	No. of Participants	Cancer Type (%)	Intervention	Follow-Up	Outcome
ACE-i/ARBs						
Nakame et al. (2005) [86]	Randomized, placebo-controlled	40	Lymphoma	Valsartan 80 mg daily	0.25 months	↓ ventricular remodeling and arrhythmia incidence LVEF unchanged in the treatment arm
Cardinale et al. (2006) [87]	Randomized, placebo-controlled	114	AML, lymphoma, MM, breast	Enalapril from dose of 2.5 mg to 20 mg daily	12 months	↓ LVEF and ↑ EDV and ESV only in untreated patients
Beta-Blockers						
Kaya et al. (2013) [88]	Double-blind, placebo-controlled	45	Breast	Nebivolol 5 mg daily	6 months	Unchanged LVESD and LVEDD in the nebivolol group ↓ declines in LVEF in the intervention arm
CECCY trial Avila et al. (2018) [89]	Double-blind, placebo-controlled	200	Breast	Carvedilol with incremental dose	6 months	No impact of carvedilol in LVEF reduction
Beta-Blockers + ACE-i/ARBs						
OVERCOME trial Bosch X et al. (2013) [90]	Double-blind, placebo-controlled	90	ALL, AML, lymphoma, MM	Enalapril + carvedilol	6 months	LVEF unchanged in the intervention group while significantly ↓ in controls
PRADA trial Heck et al. (2021) [91]	2 × 2 factorial, randomized-placebo controlled trial	120	Breast	Metoprolol combined candesartan	23 months	Candesartan + metoprolol > no change in LVEF, GLS, or LVESD candesartan alone > ↓ declines in GLS and EDV [91,92]
MRAs						
ELEVATE trial Davis et al. (2019) [93]	Randomized placebo-controlled trial	44	Breast	Eplerenone 50 mg daily	6 months	No significant differences in LV systolic or diastolic dysfunction were observed compared to the placebo group
Statins						
PREVENT trial Hundley et al. (2022) [94]	Double-blind, placebo-controlled	279	Breast (85%), lymphoma (15%)	Atorvastatin 40 mg daily	24 months	No difference in final LVEF, adjusted for baseline
SPARE-HF trial Thavendiranathan et al. (2023) [95]	Double-blind, placebo-controlled	112	Breast (65%), lymphoma (21%), sarcoma (6%), thymoma (5%), leukemia (3%)	Atorvastatin 40 mg daily	2.5 months	No difference in final LVEF, adjusted for baseline
STOP-CA trial Neilan et al. (2023) [96]	Double-blind, placebo-controlled	300	Lymphoma (100%)	Atorvastatin 40 mg daily	12 months	↓ incidence of CTRCD in the statin arm

**Table 2:** HFA-ICOS tool.

Patient-Specific Factors	
Pre-existing cardiovascular conditions	Hypertension, diabetes, hyperlipidemia, coronary artery disease, or previous heart failure.
Age	Older age increases susceptibility to cardiotoxicity due to general decline in cardiac function with aging.
Genetic predisposition	Specific genetic factors can increase sensitivity to chemotherapy-induced heart damage.
Gender	Gender-related differences may exist, with studies showing women may be at higher risk.
Lifestyle factors	Smoking, sedentary lifestyle, and poor dietary habits can increase cardiovascular risk.
Treatment-Specific Factors	
Type and cumulative dose of chemotherapy agents	Anthracyclines (like doxorubicin) and trastuzumab are cardiotoxic, especially at higher cumulative doses.
Radiotherapy	Chest irradiation increases the risk, particularly for left-sided breast cancer treatments.
Combination therapies	Some chemotherapy regimens, when used with other medications that affect cardiac function, may increase the risk.
Baseline Cardiac Function	
Left ventricular ejection fraction (LVEF)	Lower baseline LVEF can indicate higher risk of developing significant cardiotoxicity.
Cancer-Specific Factors	
Type of cancer	Certain cancers (e.g., breast cancer or hematologic malignancies) carry different risks for chemotherapy-induced cardiac damage.
Stage of cancer	The stage of cancer and its treatment protocol impact the risk of cardiotoxicity.

## Data Availability

Not applicable.

## References

[1] Lotrionte M., Biondi-Zoccai G., Abbate A., Lanzetta G., D’Ascenzo F., Malavasi V., Peruzzi M., Frati G., Palazzoni G. (2013). Review and meta-analysis of incidence and clinical predictors of anthracycline cardiotoxicity. Am. J. Cardiol..

[2] Lyon A.R., Lopez-Fernandez T., Couch L.S., Asteggiano R., Aznar M.C., Bergler-Klein J., Boriani G., Cardinale D., Cordoba R., Cosyns B. (2022). 2022 ESC Guidelines on cardio-oncology developed in collaboration with the European Hematology Association (EHA), the European Society for Therapeutic Radiology and Oncology (ESTRO) and the International Cardio-Oncology Society (IC-OS). Eur. Heart J.-Cardiovasc. Imaging.

[3] Camilli M., Cipolla C.M., Dent S., Minotti G., Cardinale D.M. (2024). Anthracycline Cardiotoxicity in Adult Cancer Patients: JACC: CardioOncology State-of-the-Art Review. JACC CardioOncolgy.

[4] Fabiani I., Chianca M., Cipolla C.M., Cardinale D.M. (2025). Anthracycline-induced cardiomyopathy: Risk prediction, prevention and treatment. Nat. Rev. Cardiol..

[5] Fabiani I., Chianca M., Aimo A., Emdin M., Dent S., Fedele A., Cipolla C.M., Cardinale D.M. (2024). Use of new and emerging cancer drugs: What the cardiologist needs to know. Eur. Heart J..

[6] Balough E., Ariza A., Asnani A., Hoeger C.W. (2025). Cardiotoxicity of Anthracyclines. Cardiol. Clin..

[7] Iervolino A., Spadafora L., Spadaccio C., Iervolino V., Biondi Zoccai G., Andreotti F. (2022). Myocardial Cell Preservation from Potential Cardiotoxic Drugs: The Role of Nanotechnologies. Pharmaceutics.

[8] Lyon A.R., Dent S., Stanway S., Earl H., Brezden-Masley C., Cohen-Solal A., Tocchetti C.G., Moslehi J.J., Groarke J.D., Bergler-Klein J. (2020). Baseline cardiovascular risk assessment in cancer patients scheduled to receive cardiotoxic cancer therapies: A position statement and new risk assessment tools from the Cardio-Oncology Study Group of the Heart Failure Association of the European Society of Cardiology in collaboration with the International Cardio-Oncology Society. Eur. J. Heart Fail..

[9] Cardinale D., Sandri M.T., Martinoni A., LabTech A.T., Civelli M., Lamantia G., Cinieri S., Martinelli G., Cipolla C.M., Fiorentini C. (2000). Left ventricular dysfunction predicted by early troponin I release after high-dose chemotherapy. J. Am. Coll. Cardiol..

[10] Cardinale D., Colombo A., Bacchiani G., Tedeschi I., Meroni C.A., Veglia F., Civelli M., Lamantia G., Colombo N., Curigliano G. (2015). Early detection of anthracycline cardiotoxicity and improvement with heart failure therapy. Circulation.

[11] Bansal N., Amdani S., Lipshultz E.R., Lipshultz S.E. (2017). Chemotherapy-induced cardiotoxicity in children. Expert Opin. Drug Metab. Toxicol..

[12] Tini G., Cuomo A., Battistoni A., Sarocchi M., Mercurio V., Ameri P., Volpe M., Porto I., Tocchetti C.G., Spallarossa P. (2022). Baseline cardio-oncologic risk assessment in breast cancer women and occurrence of cardiovascular events: The HFA/ICOS risk tool in real-world practice. Int. J. Cardiol..

[13] Camilli M., Ferdinandy P., Salvatorelli E., Menna P., Minotti G. (2024). Anthracyclines, Diastolic Dysfunction and the road to Heart Failure in Cancer survivors: An untold story. Prog. Cardiovasc. Dis..

[14] Di Lisi D., Madaudo C., Alagna G., Santoro M., Rossetto L., Siragusa S., Novo G. (2022). The new HFA/ICOS risk assessment tool to identify patients with chronic myeloid leukaemia at high risk of cardiotoxicity. ESC Heart Fail..

[15] Giallauria F., Vitelli A., Maresca L., De Magistris M.S., Chiodini P., Mattiello A., Gentile M., Mancini M., Grieco A., Russo A. (2016). Exercise training improves cardiopulmonary and endothelial function in women with breast cancer: Findings from the Diana-5 dietary intervention study. Intern. Emerg. Med..

[16] Curigliano G., Lenihan D., Fradley M., Ganatra S., Barac A., Blaes A., Herrmann J., Porter C., Lyon A.R., Lancellotti P. (2020). Management of cardiac disease in cancer patients throughout oncological treatment: ESMO consensus recommendations. Ann. Oncol..

[17] Oikonomou E.K., Kokkinidis D.G., Kampaktsis P.N., Amir E.A., Marwick T.H., Gupta D., Thavendiranathan P. (2019). Assessment of Prognostic Value of Left Ventricular Global Longitudinal Strain for Early Prediction of Chemotherapy-Induced Cardiotoxicity: A Systematic Review and Meta-analysis. JAMA Cardiol..

[18] Semeraro G.C., Lamantia G., Cipolla C.M., Cardinale D. (2021). How to identify anthracycline-induced cardiotoxicity early and reduce its clinical impact in everyday practice. Kardiol. Pol..

[19] Upshaw J.N., Parson S.K., Buchsbaum R.J., Schlam I., Ruddy K.J., Durani U., Epperla N., Leong D.P. (2024). Dexrazoxane to Prevent Cardiotoxicity in Adults Treated with Anthracyclines: JACC: CardioOncology Controversies in Cardio-Oncology. JACC CardioOncology.

[20] Avagimyan A., Pogosova N., Kakturskiy L., Sheibani M., Challa A., Kogan E., Fogacci F., Mikhaleva L., Vandysheva R., Yakubovskaya M. (2024). Doxorubicin-related cardiotoxicity: Review of fundamental pathways of cardiovascular system injury. Cardiovasc. Pathol..

[21] Li H., Wang M., Huang Y. (2024). Anthracycline-induced cardiotoxicity: An overview from cellular structural perspective. Biomed. Pharmacother..

[22] Qiu Y., Jiang P., Huang Y. (2023). Anthracycline-induced cardiotoxicity: Mechanisms, monitoring, and prevention. Front. Cardiovasc. Med..

[23] Menna P., Recalcati S., Cairo G., Minotti G. (2007). An introduction to the metabolic determinants of anthracycline cardiotoxicity. Cardiovasc. Toxicol..

[24] Forrester S.J., Kikuchi D.S., Hernandes M.S., Xu Q., Griendling K.K. (2018). Reactive Oxygen Species in Metabolic and Inflammatory Signaling. Circ. Res..

[25] Carvalho R.A., Sousa R.P., Cadete V.J., Lopaschuk G.D., Palmeira C.M., Bjork J.A., Wallace K.B. (2010). Metabolic remodeling associated with subchronic doxorubicin cardiomyopathy. Toxicology.

[26] Muckenthaler M.U., Galy B., Hentze M.W. (2008). Systemic iron homeostasis and the iron-responsive element/iron-regulatory protein (IRE/IRP) regulatory network. Annu. Rev. Nutr..

[27] Hanna A.D., Lam A., Tham S., Dulhunty A.F., Beard N.A. (2014). Adverse effects of doxorubicin and its metabolic product on cardiac RyR2 and SERCA2A. Mol. Pharmacol..

[28] Li H., Xia B., Chen W., Zhang Y., Gao X., Chinnathambi A., Alharbi S.A., Zhao Y. (2020). Nimbolide prevents myocardial damage by regulating cardiac biomarkers, antioxidant level, and apoptosis signaling against doxorubicin-induced cardiotoxicity in rats. J. Biochem. Mol. Toxicol..

[29] Renu K., Abilash V.G., Tirupathi Pichiah P.B., Arunachalam S. (2018). Molecular mechanism of doxorubicin-induced cardiomyopathy—An update. Eur. J. Pharmacol..

[30] Gharanei M., Hussain A., Janneh O., Maddock H.L. (2013). Doxorubicin induced myocardial injury is exacerbated following ischaemic stress via opening of the mitochondrial permeability transition pore. Toxicol. Appl. Pharmacol..

[31] An J., Li P., Li J., Dietz R., Donath S. (2009). ARC is a critical cardiomyocyte survival switch in doxorubicin cardiotoxicity. J. Mol. Med..

[32] Tewey K.M., Rowe T.C., Yang L., Halligan B.D., Liu L.F. (1984). Adriamycin-induced DNA damage mediated by mammalian DNA topoisomerase II. Science.

[33] Schirone L., Vecchio D., Valenti V., Forte M., Relucenti M., Angelini A., Zaglia T., Schiavon S., D’ambrosio L., Sarto G. (2023). MST1 mediates doxorubicin-induced cardiomyopathy by SIRT3 downregulation. Cell. Mol. Life. Sci..

[34] Schirone L., D’Ambrosio L., Forte M., Genovese R., Schiavon S., Spinosa G., Iacovone G., Valenti V., Frati G., Sciarretta S. (2022). Mitochondria and Doxorubicin-Induced Cardiomyopathy: A Complex Interplay. Cells.

[35] Christidi E., Brunham L.R. (2021). Regulated cell death pathways in doxorubicin-induced cardiotoxicity. Cell Death Dis..

[36] Nakano K., Vousden K.H. (2001). PUMA, a novel proapoptotic gene, is induced by p53. Mol. Cell.

[37] Fan X., He Y., Wu G., Chen H., Cheng X., Zhan Y., An C., Chen T., Wang X. (2023). Sirt3 activates autophagy to prevent DOX-induced senescence by inactivating PI3K/AKT/mTOR pathway in A549 cells. Biochim. Biophys. Acta (BBA) Mol. Cell Res..

[38] Li D.L., Wang Z.V., Ding G., Tan W., Luo X., Criollo A., Xie M., Jiang N., May H., Kyrychenko V. (2016). Doxorubicin Blocks Cardiomyocyte Autophagic Flux by Inhibiting Lysosome Acidification. Circulation.

[39] Tavakoli Dargani Z., Singla D.K. (2019). Embryonic stem cell-derived exosomes inhibit doxorubicin-induced TLR4-NLRP3-mediated cell death-pyroptosis. Am. J. Physiol. Heart Circ. Physiol..

[40] Sauter K.A., Wood L.J., Wong J., Iordanov M., Magun B.E. (2011). Doxorubicin and daunorubicin induce processing and release of interleukin-1beta through activation of the NLRP3 inflammasome. Cancer Biol. Ther..

[41] Meng L., Lin H., Zhang J., Lin N., Sun Z., Gao F., Luo H., Ni T., Luo W., Chi J. (2019). Doxorubicin induces cardiomyocyte pyroptosis via the TINCR-mediated posttranscriptional stabilization of NLR family pyrin domain containing 3. J. Mol. Cell. Cardiol..

[42] Zheng X., Zhong T., Ma Y., Wan X., Qin A., Yao B., Zou H., Song Y., Yin D. (2020). Bnip3 mediates doxorubicin-induced cardiomyocyte pyroptosis via caspase-3/GSDME. Life Sci..

[43] Zheng M., Kang Y.M., Liu W., Zang W.J., Bao C.Y., Qin D.N. (2012). Inhibition of cyclooxygenase-2 reduces hypothalamic excitation in rats with adriamycin-induced heart failure. PLoS ONE.

[44] Ferreira L.L., Cervantes M., Froufe H.J.C., Egas C., Cunha-Oliveira T., Sassone-Corsi P., Oliveira P.J. (2020). Doxorubicin persistently rewires cardiac circadian homeostasis in mice. Arch. Toxicol..

[45] Forte M., D’Ambrosio L., Schiattarella G.G., Salerno N., Perrone M.A., Loffredo F.S., Bertero E., Pilichou K., Manno G., Valenti V. (2024). Mitophagy modulation for the treatment of cardiovascular diseases. Eur. J. Clin. Investig..

[46] Song R., Yang Y., Lei H., Wang G., Huang Y., Xue W., Wang Y., Yao L., Zhu Y. (2018). HDAC6 inhibition protects cardiomyocytes against doxorubicin-induced acute damage by improving alpha-tubulin acetylation. J. Mol. Cell. Cardiol..

[47] Guglin M., Krischer J., Tamura R., Fink A., Bello-Matricaria L., McCaskill-Stevens W., Munster P.N. (2019). Randomized Trial of Lisinopril Versus Carvedilol to Prevent Trastuzumab Cardiotoxicity in Patients with Breast Cancer. J. Am. Coll. Cardiol..

[48] Vitale R., Marzocco S., Popolo A. (2024). Role of Oxidative Stress and Inflammation in Doxorubicin-Induced Cardiotoxicity: A Brief Account. Int. J. Mol. Sci..

[49] Garcia-Pavia P., Kim Y., Restrepo-Cordoba M.A., Lunde I.G., Wakimoto H., Smith A.M., Toepfer C.N., Getz K., Gorham J., Patel P. (2019). Genetic Variants Associated with Cancer Therapy-Induced Cardiomyopathy. Circulation.

[50] Hahn V.S., Zhang K.W., Sun L., Narayan V., Lenihan D.J., Ky B. (2021). Heart Failure with Targeted Cancer Therapies: Mechanisms and Cardioprotection. Circ. Res..

[51] Armenian S.H., Lacchetti C., Lenihan D. (2017). Prevention and Monitoring of Cardiac Dysfunction in Survivors of Adult Cancers: American Society of Clinical Oncology Clinical Practice Guideline Summary. J. Oncol. Pract..

[52] Passantino A., Dalla Vecchia L.A., Corra U., Scalvini S., Pistono M., Bussotti M., Gambarin F.I., Scrutinio D., La Rovere M.T. (2021). The Future of Exercise-Based Cardiac Rehabilitation for Patients with Heart Failure. Front. Cardiovasc. Med..

[53] Brown T.M., Pack Q.R., Aberegg E., Brewer L.C., Ford Y.R., Forman D.E., Gathright E.C., Khadanga S., Ozemek C., Thomas R.J. (2024). Core Components of Cardiac Rehabilitation Programs: 2024 Update: A Scientific Statement From the American Heart Association and the American Association of Cardiovascular and Pulmonary Rehabilitation. Circulation.

[54] Tranchita E., Murri A., Grazioli E., Cerulli C., Emerenziani G.P., Ceci R., Caporossi D., Dimauro I., Parisi A. (2022). The Beneficial Role of Physical Exercise on Anthracyclines Induced Cardiotoxicity in Breast Cancer Patients. Cancers.

[55] Naaktgeboren W.R., Binyam D., Stuiver M.M., Aaronson N.K., Teske A.J., van Harten W.H., Groen W.G., May A.M. (2021). Efficacy of Physical Exercise to Offset Anthracycline-Induced Cardiotoxicity: A Systematic Review and Meta-Analysis of Clinical and Preclinical Studies. J. Am. Heart. Assoc..

[56] Ghignatti P., Nogueira L.J., Lehnen A.M., Leguisamo N.M. (2021). Cardioprotective effects of exercise training on doxorubicin-induced cardiomyopathy: A systematic review with meta-analysis of preclinical studies. Sci. Rep..

[57] Pahlavani H.A. (2022). Exercise-induced signaling pathways to counteracting cardiac apoptotic processes. Front. Cell Dev. Biol..

[58] Schuttler D., Clauss S., Weckbach L.T., Brunner S. (2019). Molecular Mechanisms of Cardiac Remodeling and Regeneration in Physical Exercise. Cells.

[59] Jiang J., Ni L., Zhang X., Chatterjee E., Lehmann H.I., Li G., Xiao J. (2023). Keeping the Heart Healthy: The Role of Exercise in Cardiac Repair and Regeneration. Antioxid. Redox Signal..

[60] Nijholt K.T., Sanchez-Aguilera P.I., Voorrips S.N., de Boer R.A., Westenbrink B.D. (2022). Exercise: A molecular tool to boost muscle growth and mitochondrial performance in heart failure?. Eur. J. Heart Fail..

[61] Howden E.J., Bigaran A., Beaudry R., Fraser S., Selig S., Foulkes S., Antill Y., Nightingale S., Loi S., Haykowsky M.J. (2019). Exercise as a diagnostic and therapeutic tool for the prevention of cardiovascular dysfunction in breast cancer patients. Eur. J. Prev. Cardiol..

[62] Dimeo F., Pagonas N., Seibert F., Arndt R., Zidek W., Westhoff T.H. (2012). Aerobic exercise reduces blood pressure in resistant hypertension. Hypertension.

[63] Lu L., Mei D.F., Gu A.G., Wang S., Lentzner B., Gutstein D.E., Zwas D., Homma S., Yi G.-H., Wang J. (2002). Exercise training normalizes altered calcium-handling proteins during development of heart failure. J. Appl. Physiol..

[64] Schmitz K.H., Courneya K.S., Matthews C., Demark-Wahnefried W., Galvão D.A., Pinto B.M., Irwin M.L., Wolin K.Y., Segal R.J., Lucia A. (2010). American College of Sports Medicine roundtable on exercise guidelines for cancer survivors. Med. Sci. Sports Exerc..

[65] Adams V., Linke A. (2019). Impact of exercise training on cardiovascular disease and risk. Biochim. Biophys. Acta (BBA) Mol. Basis Dis..

[66] Campbell K.L., Winters-Stone K.M., Wiskemann J., May A.M., Schwartz A.L., Courneya K.S., Zucker D.S., Matthews C.E., Ligibel J.A., Gerber L.H. (2019). Exercise Guidelines for Cancer Survivors: Consensus Statement from International Multidisciplinary Roundtable. Med. Sci. Sports Exerc..

[67] Gilchrist S.C., Barac A., Ades P.A., Alfano C.M., Franklin B.A., Jones L.W., La Gerche A., Ligibel J.A., Lopez G., Madan K. (2019). Cardio-Oncology Rehabilitation to Manage Cardiovascular Outcomes in Cancer Patients and Survivors: A Scientific Statement From the American Heart Association. Circulation.

[68] Herranz-Gomez A., Cuenca-Martinez F., Suso-Marti L., Varangot-Reille C., Calatayud J., Blanco-Díaz M., Casaña J. (2022). Effectiveness of HIIT in patients with cancer or cancer survivors: An umbrella and mapping review with meta-meta-analysis. Scand. J. Med. Sci. Sports.

[69] Ligibel J.A., Bohlke K., May A.M., Clinton S.K., Demark-Wahnefried W., Gilchrist S.C., Irwin M.L., Late M., Mansfield S., Marshall T.F. (2022). Exercise, Diet, and Weight Management During Cancer Treatment: ASCO Guideline. J. Clin. Oncol..

[70] Kang D.W., Wilson R.L., Christopher C.N., Normann A.J., Barnes O., Lesansee J.D., Choi G., Dieli-Conwright C.M. (2021). Exercise Cardio-Oncology: Exercise as a Potential Therapeutic Modality in the Management of Anthracycline-Induced Cardiotoxicity. Front. Cardiovasc. Med..

[71] Frazelle M.L., Friend P.J. (2016). Optimizing the Teachable Moment for Health Promotion for Cancer Survivors and Their Families. J. Adv. Pract. Oncol..

[72] Shephard R.J. (2009). Maximal oxygen intake and independence in old age. Br. J. Sports Med..

[73] Stephenson E., McLaughlin M., Bray J.W., Saxton J.M., Vince R.V. (2024). Nutrition Modulation of Cardiotoxicity in Breast Cancer: A Scoping Review. Nutrients.

[74] Saini R. (2011). Coenzyme Q10: The essential nutrient. J. Pharm. Bioallied Sci..

[75] Mortensen S.A., Rosenfeldt F., Kumar A., Dolliner P., Filipiak K.J., Pella D., Alehagen U., Steurer G., Littarru G.P. (2014). The effect of coenzyme Q10 on morbidity and mortality in chronic heart failure: Results from Q-SYMBIO: A randomized double-blind trial. JACC Heart Fail..

[76] Rabanal-Ruiz Y., Llanos-Gonzalez E., Alcain F.J. (2021). The Use of Coenzyme Q10 in Cardiovascular Diseases. Antioxidants.

[77] Kumar A., Kaur H., Devi P., Mohan V. (2009). Role of coenzyme Q10 (CoQ10) in cardiac disease, hypertension and Meniere-like syndrome. Pharmacol. Ther..

[78] Weant K.A., Smith K.M. (2005). The role of coenzyme Q10 in heart failure. Ann. Pharmacother..

[79] Nonn L., Peng L., Feldman D., Peehl D.M. (2006). Inhibition of p38 by vitamin D reduces interleukin-6 production in normal prostate cells via mitogen-activated protein kinase phosphatase 5: Implications for prostate cancer prevention by vitamin D. Cancer Res..

[80] Zittermann A., Schleithoff S.S., Koerfer R. (2005). Putting cardiovascular disease and vitamin D insufficiency into perspective. Br. J. Nutr..

[81] Lee K.J., Wright G., Bryant H., Wiggins L.A., Zotto V.L.D., Schuler M., Malozzi C., Cohen M.V., Gassman N.R. (2021). Cytoprotective Effect of Vitamin D on Doxorubicin-Induced Cardiac Toxicity in Triple Negative Breast Cancer. Int. J. Mol. Sci..

[82] Widmer R.J., Flammer A.J., Lerman L.O., Lerman A. (2015). The Mediterranean diet, its components, and cardiovascular disease. Am. J. Med..

[83] Ramirez M.U., Clear K.Y.J., Cornelius Z., Bawaneh A., Feliz-Mosquea Y.R., Wilson A.S., Ruggiero A.D., Cruz-Diaz N., Shi L., Kerr B.A. (2022). Diet impacts triple-negative breast cancer growth, metastatic potential, chemotherapy responsiveness, and doxorubicin-mediated cardiac dysfunction. Physiol. Rep..

[84] Visseren F.L.J., Mach F., Smulders Y.M., Carballo D., Koskinas K.C., Bäck M., Benetos A., Biffi A., Boavida J.-M., Capodanno D. (2022). 2021 ESC Guidelines on cardiovascular disease prevention in clinical practice: Developed by the Task Force for cardiovascular disease prevention in clinical practice with representatives of the European Society of Cardiology and 12 medical societies with the special contribution of the European Association of Preventive Cardiology (EAPC). Rev. Esp. Cardiol..

[85] Adams S.C., Rivera-Theurel F., Scott J.M., Nadler M.B., Foulkes S., Leong D., Nilsen T., Porter C., Haykowsky M., Abdel-Qadir H. (2025). Cardio-oncology rehabilitation and exercise: Evidence, priorities, and research standards from the ICOS-CORE working group. Eur. Heart J..

[86] Nakamae H., Tsumura K., Terada Y., Nakane T., Nakamae M., Ohta K., Yamane T., Hino M. (2005). Notable effects of angiotensin II receptor blocker, valsartan, on acute cardiotoxic changes after standard chemotherapy with cyclophosphamide, doxorubicin, vincristine, and prednisolone. Cancer.

[87] Cardinale D., Colombo A., Sandri M.T., Lamantia G., Colombo N., Civelli M., Martinelli G., Veglia F., Fiorentini C., Cipolla C.M. (2006). Prevention of high-dose chemotherapy-induced cardiotoxicity in high-risk patients by angiotensin-converting enzyme inhibition. Circulation.

[88] Kaya M.G., Ozkan M., Gunebakmaz O., Akkaya H., Kaya E.G., Akpek M., Kalay N., Dikilitas M., Yarlioglues M., Karaca H. (2013). Protective effects of nebivolol against anthracycline-induced cardiomyopathy: A randomized control study. Int. J. Cardiol..

[89] Avila M.S., Ayub-Ferreira S.M., de Barros Wanderley M.R., Cruz F.d.D., Brandão S.M.G., Rigaud V.O.C., Higuchi-Dos-Santos M.H., Hajjar L.A., Filho R.K., Hoff P.M. (2018). Carvedilol for Prevention of Chemotherapy-Related Cardiotoxicity: The CECCY Trial. J. Am. Coll. Cardiol..

[90] Bosch X., Rovira M., Sitges M., Domènech A., Ortiz-Pérez J.T., de Caralt T.M., Morales-Ruiz M., Perea R.J., Monzó M., Esteve J. (2013). Enalapril and carvedilol for preventing chemotherapy-induced left ventricular systolic dysfunction in patients with malignant hemopathies: The OVERCOME trial (preventiOn of left Ventricular dysfunction with Enalapril and caRvedilol in patients submitted to intensive ChemOtherapy for the treatment of Malignant hEmopathies). J. Am. Coll. Cardiol..

[91] Heck S.L., Mecinaj A., Ree A.H., Hoffmann P., Schulz-Menger J.E., Fagerland M.W., Gravdehaug B., Røsjø H., Steine K., Geisler J. (2021). Prevention of Cardiac Dysfunction During Adjuvant Breast Cancer Therapy (PRADA): Extended Follow-Up of a 2x2 Factorial, Randomized, Placebo-Controlled, Double-Blind Clinical Trial of Candesartan and Metoprolol. Circulation.

[92] Gulati G., Heck S.L., Ree A.H., Hoffmann P., Schulz-Menger J., Fagerland M.W., Gravdehaug B., von Knobelsdorff-Brenkenhoff F., Bratland Å., Storås T.H. (2016). Prevention of cardiac dysfunction during adjuvant breast cancer therapy (PRADA): A 2 × 2 factorial, randomized, placebo-controlled, double-blind clinical trial of candesartan and metoprolol. Eur. Heart J..

[93] Davis M.K., Villa D., Tsang T.S.M., Starovoytov A., Gelmon K., Virani S.A. (2019). Effect of Eplerenone on Diastolic Function in Women Receiving Anthracycline-Based Chemotherapy for Breast Cancer. JACC CardioOncology.

[94] Hundley W.G., D’Agostino R., Crotts T., Craver K., Hackney M.H., Jordan J.H., Ky B., Wagner L.I., Herrington D.M., Yeboah J. (2022). Statins and Left Ventricular Ejection Fraction Following Doxorubicin Treatment. NEJM Evid..

[95] Thavendiranathan P., Houbois C., Marwick T.H., Kei T., Saha S., Runeckles K., Huang F., Shalmon T., Thorpe K.E., Pezo R.C. (2023). Statins to prevent early cardiac dysfunction in cancer patients at increased cardiotoxicity risk receiving anthracyclines. Eur. Heart J. Cardiovasc. Pharmacother..

[96] Neilan T.G., Quinaglia T., Onoue T., Mahmood S.S., Drobni Z.D., Gilman H.K., Smith A., Heemelaar J.C., Brahmbhatt P., Ho J.S. (2023). Atorvastatin for Anthracycline-Associated Cardiac Dysfunction: The STOP-CA Randomized Clinical Trial. JAMA.

[97] Sobczuk P., Czerwinska M., Kleibert M., Cudnoch-Jedrzejewska A. (2022). Anthracycline-induced cardiotoxicity and renin-angiotensin-aldosterone system-from molecular mechanisms to therapeutic applications. Heart Fail. Rev..

[98] Bozcali E., Dedeoglu D.B., Karpuz V., Suzer O., Karpuz H. (2012). Cardioprotective effects of zofenopril, enalapril and valsartan against ischaemia/reperfusion injury as well as doxorubicin cardiotoxicity. Acta Cardiol..

[99] Dessi M., Madeddu C., Piras A., Cadeddu C., Antoni G., Mercuro G., Mantovani G. (2013). Long-term, up to 18 months, protective effects of the angiotensin II receptor blocker telmisartan on Epirubin-induced inflammation and oxidative stress assessed by serial strain rate. Springerplus.

[100] Lother A., Bergemann S., Kowalski J., Huck M., Gilsbach R., Bode C., Hein L. (2018). Inhibition of the cardiac myocyte mineralocorticoid receptor ameliorates doxorubicin-induced cardiotoxicity. Cardiovasc. Res..

[101] Oesterle A., Laufs U., Liao J.K. (2017). Pleiotropic Effects of Statins on the Cardiovascular System. Circ. Res..

[102] Rashid M., Tawara S., Fukumoto Y., Seto M., Yano K., Shimokawa H. (2009). Importance of Rac1 signaling pathway inhibition in the pleiotropic effects of HMG-CoA reductase inhibitors. Circ. J..

[103] Maack C., Kartes T., Kilter H., Schafers H.-J., Nickenig G., Bohm M., Laufs U. (2003). Oxygen free radical release in human failing myocardium is associated with increased activity of rac1-GTPase and represents a target for statin treatment. Circulation.

[104] Huelsenbeck J., Henninger C., Schad A., Lackner K.J., Kaina B., Fritz G. (2011). Inhibition of Rac1 signaling by lovastatin protects against anthracycline-induced cardiac toxicity. Cell Death Dis..

[105] Bhalraam U., Veerni R.B., Paddock S., Meng J., Piepoli M., López-Fernández T., Tsampasian V., Vassiliou V.S. (2025). Impact of sodium-glucose cotransporter-2 inhibitors on heart failure outcomes in cancer patients and survivors: A systematic review and meta-analysis. Eur. J. Prev. Cardiol..

[106] Gongora C.A., Drobni Z.D., Silva T.Q.A.C., Zafar A., Gong J., Zlotoff D.A., Gilman H.K., Hartmann S.E., Sama S., Nikolaidou S. (2022). Sodium-Glucose Co-Transporter-2 Inhibitors and Cardiac Outcomes Among Patients Treated with Anthracyclines. JACC Heart Fail..

[107] Dabour M.S., George M.Y., Daniel M.R., Blaes A.H., Zordoky B.N. (2024). The Cardioprotective and Anticancer Effects of SGLT2 Inhibitors: JACC: CardioOncology State-of-the-Art Review. JACC CardioOncology.

[108] Sabatino J., De Rosa S., Tamme L., Iaconetti C., Sorrentino S., Polimeni A., Mignogna C., Amorosi A., Spaccarotella C., Yasuda M. (2020). Empagliflozin prevents doxorubicin-induced myocardial dysfunction. Cardiovasc. Diabetol..

[109] Quagliariello V., De Laurentiis M., Rea D., Barbieri A., Monti M.G., Carbone A., Paccone A., Altucci L., Conte M., Canale M.L. (2021). The SGLT-2 inhibitor empagliflozin improves myocardial strain, reduces cardiac fibrosis and pro-inflammatory cytokines in non-diabetic mice treated with doxorubicin. Cardiovasc. Diabetol..

[110] Daniele A.J., Gregorietti V., Costa D., Lopez-Fernandez T. (2024). Use of EMPAgliflozin in the prevention of CARDiotoxicity: The EMPACARD—PILOT trial. Cardio-Oncology.

[111] Li X., Luo W., Tang Y., Wu J., Zhang J., Chen S., Zhou L., Tao Y., Tang Y., Wang F. (2024). Semaglutide attenuates doxorubicin-induced cardiotoxicity by ameliorating BNIP3-Mediated mitochondrial dysfunction. Redox. Biol..

[112] HamaSalih R.M. (2024). Effects of Semaglutide in Doxorubicin-Induced Cardiac Toxicity in Wistar Albino Rats. Cancer Manag. Res..

[113] Zeng X., Zhang H., Xu T., Mei X., Wang X., Yang Q., Luo Z., Zeng Q., Xu D., Ren H. (2024). Vericiguat attenuates doxorubicin-induced cardiotoxicity through the PRKG1/PINK1/STING axis. Transl. Res..

[114] Quagliariello V., Berretta M., Bisceglia I., Giacobbe I., Iovine M., Giordano V., Arianna R., Barbato M., Izzo F., Maurea C. (2024). The sGCa Vericiguat Exhibit Cardioprotective and Anti-Sarcopenic Effects through NLRP-3 Pathways: Potential Benefits for Anthracycline-Treated Cancer Patients. Cancers.

[115] Tajstra M., Dyrbus M., Rutkowski T., Składowski K., Sosnowska-Pasiarska B., Góźdź S., Radecka B., Staszewski M., Majsnerowska A., Myrda K. (2023). Sacubitril/valsartan for cardioprotection in breast cancer (MAINSTREAM): Design and rationale of the randomized trial. ESC Heart Fail..

[116] Hu F., Yan S., Lin L., Qiu X., Lin X., Wang W. (2025). Sacubitril/valsartan attenuated myocardial inflammation, fibrosis, apoptosis and promoted autophagy in doxorubicin-induced cardiotoxicity mice via regulating the AMPKalpha-mTORC1 signaling pathway. Mol. Cell. Biochem..

[117] Rivero-Santana B., Saldana-Garcia J., Caro-Codon J., Zamora P., Moliner P., Monzonis A.M., Zatarain E., Álvarez-Ortega C., Gómez-Prieto P., Pernas S. (2024). Anthracycline-induced cardiovascular toxicity: Validation of the Heart Failure Association and International Cardio-Oncology Society risk score. Eur. Heart J..

[118] Butel-Simoes L.E., Ngo D.T.M., Sverdlov A.L. (2025). Navigating cardiotoxicity risk in cancer therapy: The importance of the HFA-ICOS score. Eur. Heart J..

[119] Ky B., French B., Khan A.M., Plappert T., Wang A., Chirinos J.A., Fang J.C., Sweitzer N.K., Borlaug B.A., Kass D.A. (2013). Ventricular-arterial coupling, remodeling, and prognosis in chronic heart failure. J. Am. Coll. Cardiol..

[120] Saunderson C.E.D., Plein S., Manisty C.H. (2021). Role of cardiovascular magnetic resonance imaging in cardio-oncology. Eur. Heart J. Cardiovasc. Imaging.

[121] Baldassarre L.A., Ganatra S., Lopez-Mattei J., Yang E.H., Zaha V.G., Wong T.C., Ayoub C., DeCara J.M., Dent S., Deswal A. (2022). Advances in Multimodality Imaging in Cardio-Oncology: JACC State-of-the-Art Review. J. Am. Coll. Cardiol..

[122] Todorova V.K., Hsu P.C., Wei J.Y., Lopez-Candales A., Chen J.Z., Su L.J., Makhoul I. (2020). Biomarkers of inflammation, hypercoagulability and endothelial injury predict early asymptomatic doxorubicin-induced cardiotoxicity in breast cancer patients. Am. J. Cancer Res..

[123] Lee W.E., Genetzakis E., Barsha G., Vescovi J., Mifsud C., Vernon S.T., Nguyen T.V., Gray M.P., Grieve S.M., Figtree G.A. (2024). Expression of Myeloperoxidase in Patient-Derived Endothelial Colony-Forming Cells-Associations with Coronary Artery Disease and Mitochondrial Function. Biomolecules.

[124] Feng W., Wang Q., Tan Y., Qiao J., Liu Q., Yang B., Yang S., Cui L. (2025). Early detection of anthracycline-induced cardiotoxicity. Clin. Chim. Acta.

